# Frictional Wear and Thermal Fatigue Properties of Die Steel after Ultrasound-Assisted Alloying

**DOI:** 10.3390/ma16216975

**Published:** 2023-10-31

**Authors:** Chunhua Hu, Yihao Wei, Xinghao Ji, Yu Liu

**Affiliations:** 1 Key Laboratory of Automobile Materials, School of Materials Science and Engineering, Jilin University, Changchun 130025, China; huchunhua@jlu.edu.cn (C.H.); weiyh20@mails.jlu.edu.cn (Y.W.); jixh20@mails.jlu.edu.cn (X.J.); 2 School of Mechanical and Aerospace Engineering, Jilin University, Changchun 130025, China

**Keywords:** frictional wear, thermal fatigue, ultrasonic surface rolling, high-energy ion implanting

## Abstract

The surface layer of 8407 die steel was strengthened using the combination of ultrasonic surface rolling and high-energy ion implanting in the present work. The strengthened layer was then characterized via microstructure observation, composition analysis, and hardness test. After that, the frictional wear and thermal fatigue properties of high-energy ion implanting specimens and composite-reinforced specimens were compared. Results show that the pretreatment of specimens with ultrasonic surface rolling causes grain refinement in the material surface, which promotes the strengthening effect of high-energy ion implanting. The wear volume of composite-reinforced specimens at medium and high frequencies is reduced by about 20%, and the wear resistance of these specimens is significantly improved with a lower friction coefficient and wear volume at moderate and high frequencies in alternating load friction experiments. Meanwhile, the thermal fatigue crack depth of composite-reinforced specimens is reduced by about 47.5%, which effectively prevents the growth of thermal cracks in the surface, thus improving the curing ability of the implanted elements. Therefore, composite strengthening of the mold steel surface is conducive to improving the cycle life, ensuring accuracy, effectively hindering the expansion of thermal cracks, and saving the cost of production.

## 1. Introduction

Currently, molds are liable to premature failure under complex working conditions. In light of this, surface strengthening is usually adopted to extend their service life. Strengthening processes such as nitriding, laser cladding, thermal spraying, and ion implanting are commonly used to improve the hardness, wear resistance, and thermal fatigue properties of die steel [1,2,3,4]. Among these processes, high-energy ion implanting (HEII) shows superior advantages. For instance, no shedding phenomenon occurs, since the modified layer has no clear interface with the base material. Also, it does not change the original size and roughness of the workpiece, which is suitable for the final fabrication sequence of all precision parts. Although high-energy ion implanting can improve the wear resistance of various components, its implanting is fairly limited [5,6]. Considering this, surface self-nanocrystallization like shot peening, for example, is used as pretreatment. This is because grain refinement occurs after the severe plastic deformation of the material surface, accompanied by an increase in non-equilibrium defects and dislocation density, which provides more channels for ion diffusion. At the same time, shot blasting effectively improves the hardness of the original material, and the residual stress generated in the surface can prolong the fatigue life [7,8,9,10,11,12]. However, shot peening leaves pits on the material surface, leading to an increase in surface roughness, which greatly offsets the superior effects of ion implanting. It is therefore very necessary to introduce a surface self-nanocrystallization process with high precision. The ultrasonic surface rolling process (USRP) meets all of the requirements. The USRP can generate gradient nanostructure and residual compressive stress in the surface layer, as well as improve the hardness and surface smoothness, which can effectively prevent the initiation and propagation of cracks in the material surface [13].

The ultrasonic surface rolling process has been applied to modify the structural components of various complex states, such as fatigue, corrosion, and wear [14,15,16,17]. It acts on the surface of the material at a slow pace of motion to ensure uniform high-frequency impacting and micro-deformation. The combination of ultrasonic surface rolling and ion implanting will have a benign effect on each other and together obtain a satisfying strengthening effect [18,19,20]. The spherical rolling head creates microscopic grooves on the material surface during its movement and the appropriate parameters result in a high density of microstructure, which also results in an improvement in friction and wear properties [21,22].

For better understanding of the effective role of ultrasonic surface rolling on high-energy ion implanting, the ultrasonic surface rolling pretreatment followed by high-energy ion implanting of silicon nitride on the surface of 8407 die steel was carried out in this paper. Then, the friction resistance and thermal fatigue properties of the strengthened material surface were studied in detail. Relevant results will lay a foundation for the further application of the ultrasonic surface rolling process.

The contact points between the mold surface and the molding material are widely distributed, and the force during processing changes with the deformation demand of the workpiece. Therefore, it is subjected to alternating force during mold processing. Studying the friction and wear behavior of die steel under alternating load has a very practical research value. During the molding process, heat is transferred from the surface of the mold to the inside, forming a temperature gradient and resulting in the uneven heating of the mold as a whole. Under the action of the temperature gradient, the thermal deformation of the mold is not uniform. The thermal deformation of the surface is constrained, and when the thermal deformation limit is exceeded, the plastic deformation of the surface will lead to cracking, forming a thermal crack [23,24]. It has become a key research direction to solve the friction wear and thermal cracking failure of die steel surfaces in long-term use.

## 2. Materials and Methods

**Figure 1:**
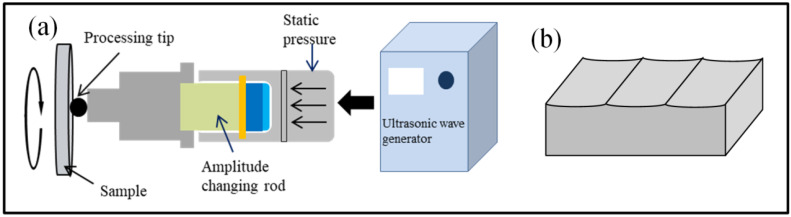
The schematic diagram of the USRP: (**a**) processing principle diagram; (**b**) shape of the rolled groove.

The material used in this study was 8407 die steel (Jiangsu Changhu Industrial Co., Ltd., Suzhou, China) in annealed conditions with the chemical composition shown in Table 1. The 8407 die steel, with an original dimension of 170 mm × 170 mm × 6 mm, was polished using 600, 800, and 1000 grit sandpaper in turn and then cleaned in anhydrous ethanol for 3 min. Partial specimens were first treated using the USRP. A HJ-III-type ultrasonic surface rolling treatment device (Tianjin University, Tianjin, China) was adopted for surface nano-treatment. During the ultrasonic surface rolling process, a 20 kHz high-frequency alternating current was generated by ultrasonic power supply, and then converted to ultrasonic mechanical vibration through a piezoelectric ceramic transducer. The mechanical vibration acted on the mold steel surface to form plastic deformation. When the USRP was performed, specimens were fixed in the spindle of lathe and the USRP tool was fixed in the feed unit of lathe. With the rotation of the spindle and the feeding of the ultrasonic tool head, the specimen surface can be treated uniformly. The processing schematic diagram of the USRP is shown in Figure 1a. The shape of the rolled groove caused by plastic deformation is schematically illustrated in Figure 1b. The processing parameters of USRP are shown in Table 2. Specimens processed using the USRP are named as USRP specimens.

The original specimens and USRP specimens were ultrasonically cleaned in anhydrous ethanol for 3 min and then placed in the HEII device (Borui Tiancheng Technology Co., Ltd., Beijing, China). All the specimens, including non-USRP-treated specimens, were pierced and hung in a vacuum injection furnace, connecting with the cathode of the furnace. SiN powder was also placed at the anode. Ionized ions were injected vertically into the specimen surface. The injection penetrant powder was placed at the source of the furnace. Test temperature was adjusted to 400–450 °C, and argon was used as the gas environment. The processing parameters of HEII are shown in Table 3.

The high-temperature environment can reduce the activation energy of the material surface, which is conducive to the depth of the ion implantation layer under high pressure. Argon is introduced into the HEII device to protect specimens from oxidation in the high-temperature environment. For simplicity, specimens are named as HEII specimens and USRP+HEII specimens, respectively. Three specimens were prepared using each of the above parameters.

These specimens were cut into small pieces, and then ground, polished, and etched sequentially to prepare for micro-observation. A 4% nitric acid alcohol solution was used for corrosion. The cross-sectional metallographic structure was observed using a Zeiss Scope Al type optical microscope (ZEISS, Oberkochen, Germany). The chemical elements were examined using scanning electron microscopy and corresponding energy spectrometry. The microhardness of the cross-section was measured using a MHVD-1000 MPa Vickers microhardness tester with an applied load of 1000 g and a holding time of 15 s. Spacing of 25 μm was implemented between every two measurement points. The measurement was repeated three times at the same depth to obtain the average value.

A D/Max 2500 PC X-ray diffractometer (Rigaku Corporation, Tokyo, Japan) was used to characterize the surface phases of specimens. The equipment utilized a Cu-Kα radiation source, which emitted X-rays with λ of 1.54156 Å. The surfaces of specimens were lightly polished with sandpaper before data acquisition. The diffraction angle was set in the range of 20–80° and the scanning rate was 0.05°/s. The measured data were processed using Jade software 6.5 to qualitatively analyze the physical phases, and to compare the diffraction peaks and half-peak widths before and after the tumbling treatment.

**Figure 2:**
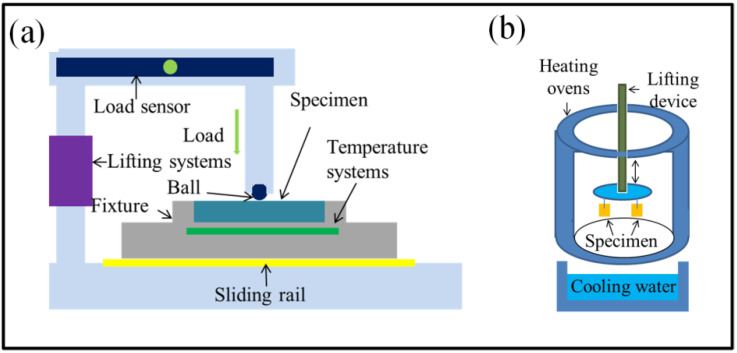
Schematic diagram of experiments for (**a**) friction and (**b**) thermal fatigue.

**Figure 3:**
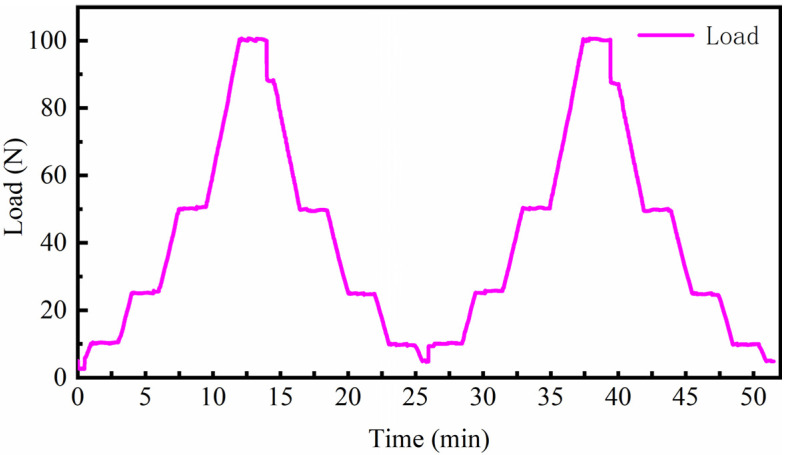
The variation of test load within one cycle.

The friction and wear properties were tested using an MFT-R4000 high-speed reciprocating friction and wear tester (CHENGXINSHIYANSHEBEI Limited Company, Zhangjiakou, China), as shown in Figure 2a. The specimen surface was lubricated by applying lubricant at a temperature of 200 °C with a frequency of 10 Hz. The variation of test load within one cycle is shown in Figure 3, and the alternating load was repeated until the end of the test.

The thermal fatigue test was performed on a thermal fatigue tester as shown in Figure 2b. Specimens were kept in a 600 °C constant-temperature oven for 225 s and then cooled in 25 °C flowing water for 5 s. The time it took to transfer from the oven to the flowing water was 10 s. This cycle was repeated 1920 times.

## 3. Results and Discussion

### 3.1. Surface Characterization

**Figure 4:**
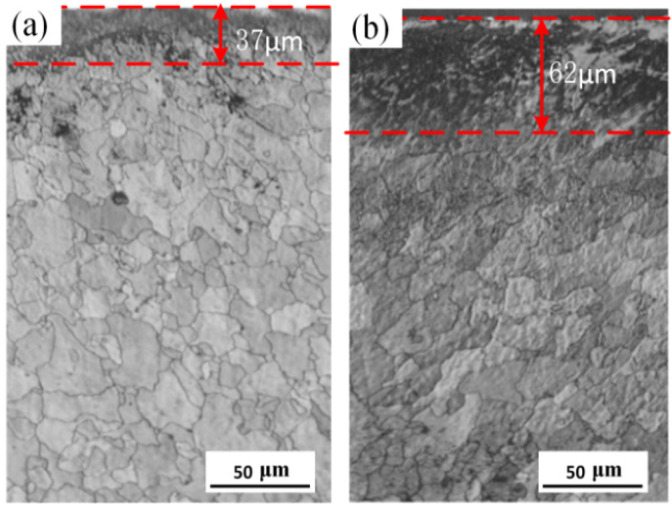
The microstructure of cross-section for: (**a**) HEII, and (**b**) USRP+HEII.

The cross-sectional microstructures of the HEII specimen and the USRP+HEII specimen are shown in Figure 4. As can be seen, the metallographic structure is mainly composed of austenite and ferrite in both types of specimens. The Si and N elements hit the surface of the specimen with high kinetic energy, resulting in a deeper color compared to that of the matrix. The thickness of the deeper color layer of the HEII specimen and USRP+HEII specimen is about 37 μm and 62 μm, respectively. Clearly, the USRP+HEII specimen has better elemental enrichment. The combined rolling and ultrasonic peening leads to an increase in grain boundaries and other microscopic defects, which facilitate the diffusion process of infiltrated elements from the specimen surface into the matrix, thus presenting a deep injection layer.

**Figure 5:**
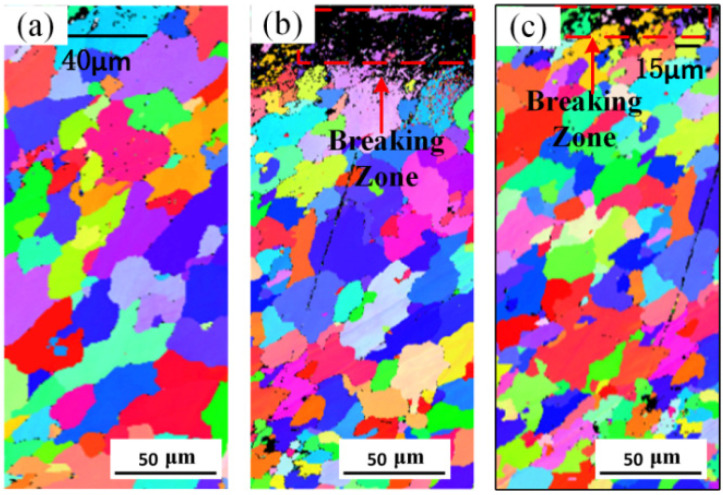
Cross-sectional EBSD images for: (**a**) HEII specimen, (**b**) USRP specimen, and (**c**) USRP+HEII specimen.

The EBSD images of the HEII specimen and USRP+HEII specimen are shown in Figure 5. It can be seen from Figure 5a that grains in the surface of the HEII specimen remain the original size of about 40 μm. In Figure 5b, it can be observed that the undistinguished region in the material surface after USRP treatment is larger and densely distributed with more point-like regions, which indicates that the region undergoes denser plastic deformation with higher grain refinement, and the depth of the deformed region is about 25 μm. The surface grains are randomly oriented and refined to nanoscale. While in Figure 5c, comparing with the USRP sample, the grains in the surface of the USRP+HEII sample have grown under the high-temperature environment of energetic ion impregnation. But the size of grains is much smaller than those in the HEII sample, and the grown grains still contribute to the improvement of the effect of energetic ion impregnation. The area of grain refinement (in the red dashed box) is about 20 μm deep from the surface. The grains below this area are about 15 μm in size and gradually coarsen to their original size at a depth of 150 μm from the surface.

**Figure 6:**
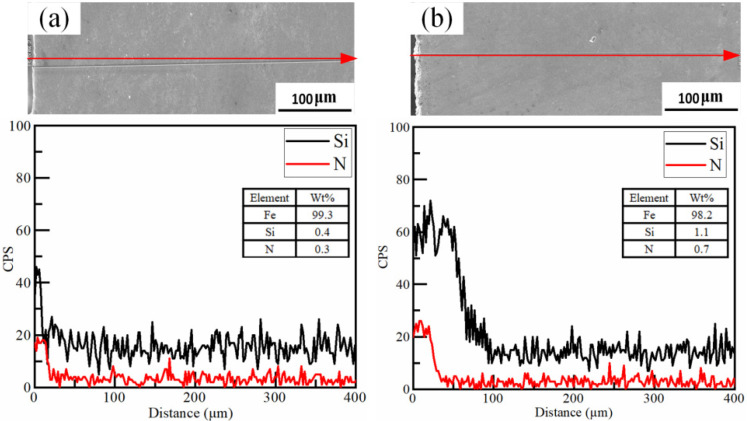
Cross-sectional line scanning results for: (**a**) HEII specimen, and (**b**) USRP+HEII specimen (red arrows represent the depth direction).

The cross-sectional line scanning results of the HEII specimen and USRP+HEII specimen are shown in Figure 6. The signal intensities of Si and N are obviously larger at the surface of both types of specimens. As shown in Figure 6a, the intensified Si signal and N signal are detected as deep as 20 μm below the surface of the HEII specimen. While for the USRP+HEII specimen in Figure 6b, the intensity of the Si element is improved more dramatically and deeply, up to 70 μm below the surface. The N signal shows the same trend, but the increase in strength and depth is smaller. This can be explained by the diffusion of elements deeper into the specimen due to dislocations in the surface of the USRP specimen, which effectively increase the thickness of the strengthened layer. The mass percent ratios of elemental Si and N for the two specimens show that the USRP+HEII specimen has better high-energy ion implanting.

**Figure 7:**
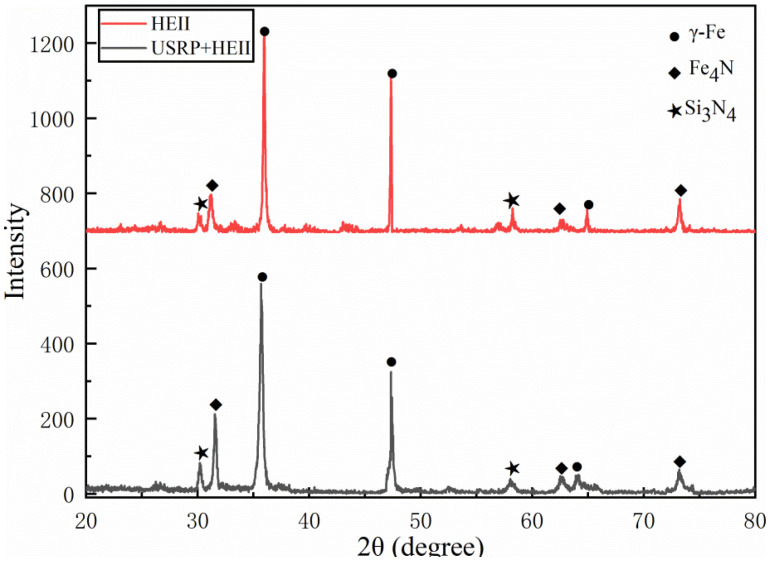
X-ray diffraction patterns of HEII-treated surface and USRP+HEII-treated surface.

The X-ray diffraction patterns of the HEII-treated surface and USRP+HEII-treated surface are shown in Figure 7. The γ-Fe, Fe_3_N, and Si_3_N_4_ phases appear on the surfaces of both the HEII specimen and the USRP+HEII specimen, reflecting the fact that the presence of reinforced phases is mainly in the form of iron compounds and silicon nitride. Compared with the HEII specimen, the diffraction intensity of Fe_3_N and Si_3_N_4_ in the USRP+HEII specimen has increased and the diffraction peaks have broadened to different degrees.

The half-height width values of the γ-Fe diffraction peak of the HEII specimens and USRP+HEII specimens are shown in Table 4. γ-Fe is the matrix phase of 8407 die steel. Again, it can be seen from Table 4 that the half-height width of the matrix phase is significantly broadened after USRP treatment, indicating that severe plastic deformation occurs on the specimen surface after the USRP [25].

**Figure 8:**
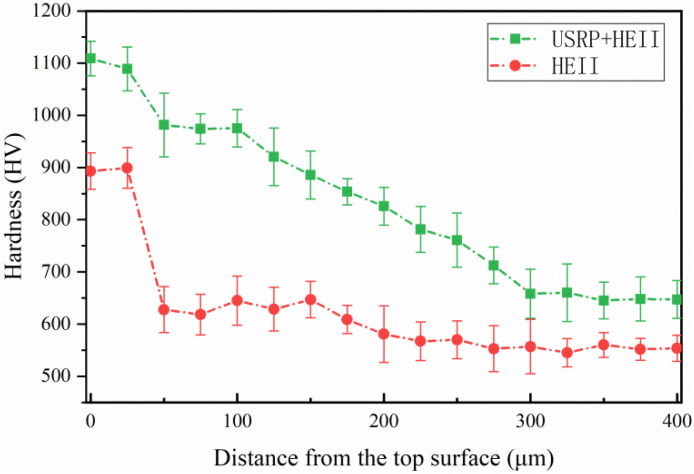
Cross-sectional microhardness distribution of HEII specimens and USRP+HEII specimens.

The cross-sectional microhardness values of the HEII specimen and USRP+HEII specimen are shown in Figure 8. The surface hardness of the USRP+HEII specimen is increased by 22.2% compared to that of the HEII specimen, and the average hardness of the USRP+HEII specimen is 19.8% higher than that of the HEII specimen within 250 μm from the surface. This is because the apparent increased hardness after the USRP is further increased through HEII. Moreover, the diffusion concentration and depth of silicon nitride are both improved by the pretreatment of the USRP, which is more conducive to the improvement of hardness.

### 3.2. Friction and Wear Properties

**Figure 9:**
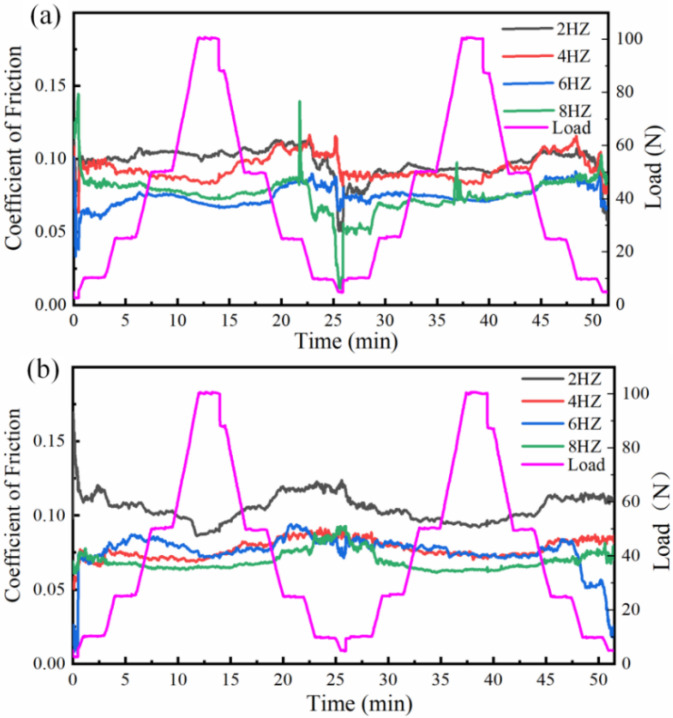
Friction coefficients at different frequencies under alternating load: (**a**) HEII, and (**b**) USRP+HEII.

Figure 9 shows the variation of the friction coefficient for the HEII specimens and USRP+HEII specimens at different friction frequencies with alternating friction loads. The friction coefficient of the HEII specimens and USRP+HEII specimens decreases when the load increases and increases when the load decreases. The USRP+HEII specimens have a higher surface hardness than HEII specimens, and support pressure changes better when the load changes, so their friction coefficient is more stable. The USRP+HEII specimens have smaller and more stable friction coefficients at 4 Hz, 6 Hz, and 8 Hz.

**Figure 10:**
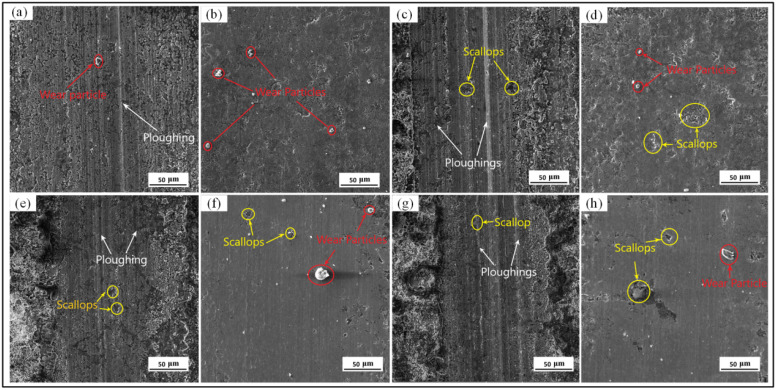
Worn morphologies of HEII specimens and USRP+HEII specimens at different frequencies: (**a**) HEII specimen at 2 Hz, (**b**) USRP+HEII specimen at 2 Hz, (**c**) HEII specimen at 4 Hz, (**d**) USRP+HEII specimen at 4 Hz, (**e**) HEII specimen at 6 Hz, (**f**) USRP+HEII specimen at 6 Hz, (**g**) HEII specimen at 8 Hz, and (**h**) USRP+HEII specimen at 8 Hz.

The morphologies of the HEII specimens at different frequencies are shown in Figure 10a,c,e,g. The reciprocal motion of hard particles produced more furrows on the surface of the HEII specimens, making the surface uneven. As the frequency of friction increases, the density of furrows gradually increases. The HEII specimen has a low bond strength between the percolation layer and the substrate, which causes flaking and detachment under the pressure of reciprocal friction to form abrasive particles which are dispersed on the surface of abrasion marks. The wear mechanism of the HEII specimen can be concluded as aggressive abrasive and adhesive wear. Figure 10b,d,f,h show the wear morphologies of the USRP+HEII specimens at different frequencies. Dislodged abrasive grains, fine furrows, and shallow spalling pits can be observed on these surfaces, indicating that the USRP+HEII specimens have good enough strength and bonding properties to cope well with high-frequency reciprocating friction movements. The USRP+HEII specimens present good resistance to abrasive wear with slight adhesive wear.

**Figure 11:**
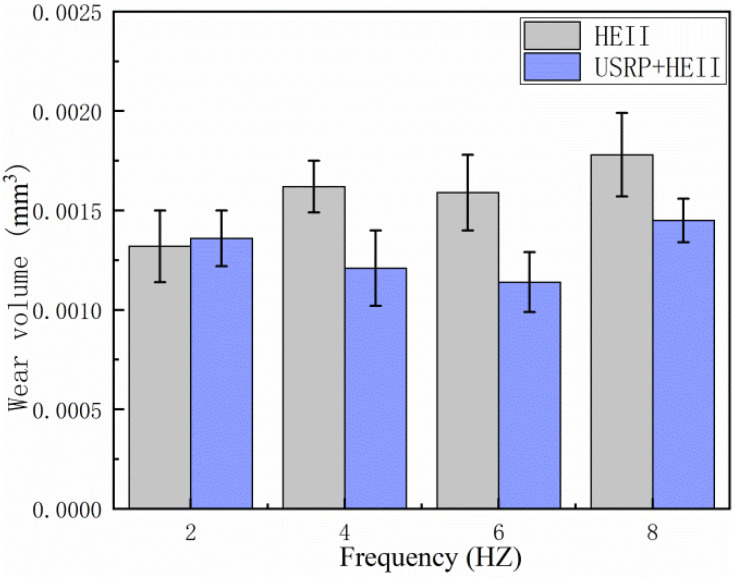
Wear volume of HEII specimens and USRP+HEII specimens at different frequencies.

Figure 11 shows the wear volumes of the HEII specimen and USRP+HEII specimens at different frequencies. Clearly, the wear volume of the HEII specimen is higher than that of the USRP+HEII specimen at each frequency. The values for the USRP+HEII specimens at 4 Hz and 6 Hz can be maintained at a lower level, indicating that the USRP+HEII specimens have better wear resistance under these wear conditions. No significant difference can be found between the HEII specimens and USRP+HEII specimens at 2 Hz. The USRP+HEII specimens show a 25%, 28.3%, and 18.5% reduction in wear volume at frequencies greater than 2 Hz, respectively, and by comparison, it can be concluded that the USRP+HEII specimens have a smaller wear volume.

### 3.3. Thermal Fatigue

**Figure 12:**
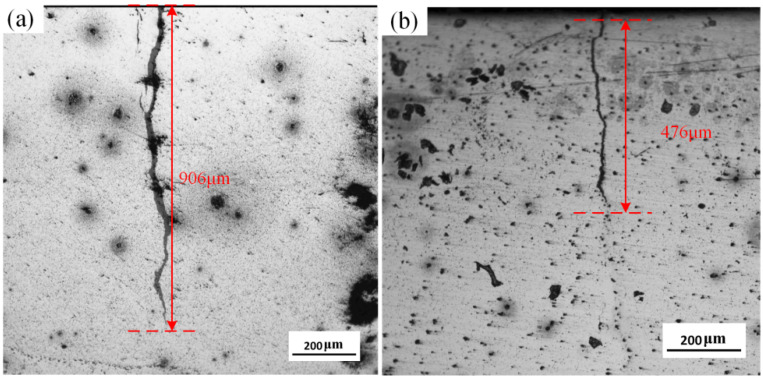
Thermal fatigue cracking of specimens: (**a**) HEII, and (**b**) USRP+HEII.

The thermal fatigue cracks of the HEII specimens and USRP+HEII specimens are shown in Figure 12. The crack length of the HEII specimen is about 906 μm. It is wider in the initial stage and gradually becomes narrower as the crack grows. The crack length of the USRP+HEII specimen is about 476 μm, which is approximately 47.5% shorter than that of the HEII specimen, and the crack is distinctly narrower compared with that of the HEII specimen. The crack propagation direction of the USRP+HEII specimen changes several times, indicating that the crack is blocked several times during propagation. The refined grains generated through USRP treatment and the increased hardness after HEII treatment both improve the thermal crack resistance [26,27].

**Figure 13:**
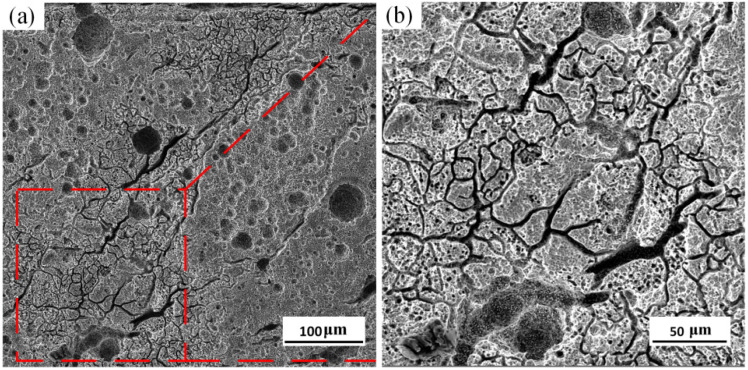
Surface morphology of HEII specimen: (**a**) representative region; (**b**) enlarged view of the red dotted box.

The surface morphology of the HEII specimen is shown in Figure 13. As can be seen, dense cracks and pits distribute in the surface. The maximum width of crack reaches 10 μm, and the maximum diameter of pit is about 50 μm. The material spalling in the specimen surface is relatively serious. It can be observed that the primary cracks in the specimen surface are deeper. The secondary cracks propagate from the substrate of the primary cracks and densely distribute across the whole surface of the specimen. The overall damage is relatively large.

**Figure 14:**
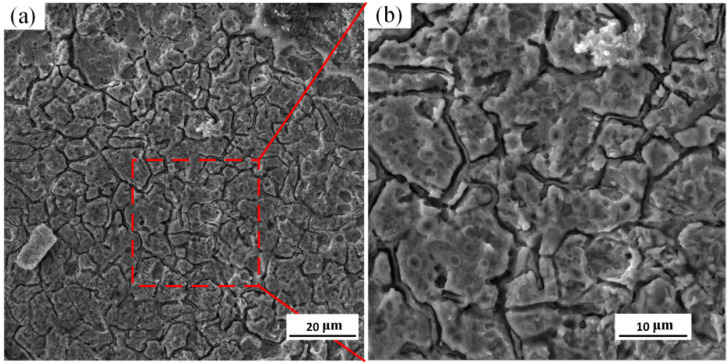
Surface morphology of USRP+HEII specimen: (**a**) representative region; (**b**) enlarged view of the red dotted box.

Figure 14 shows the surface morphology of the USRP+HEII specimen. The cracks are relatively small and narrow, and evenly distributed across the surface without obvious pit spalling. The grain refinement and high strength of the USRP+HEII specimens effectively inhibit the propagation of thermal cracks, and the better bonding strength ensures the surface integrity of specimens. Thus, the specimens have slight surface failure [28].

**Figure 15:**
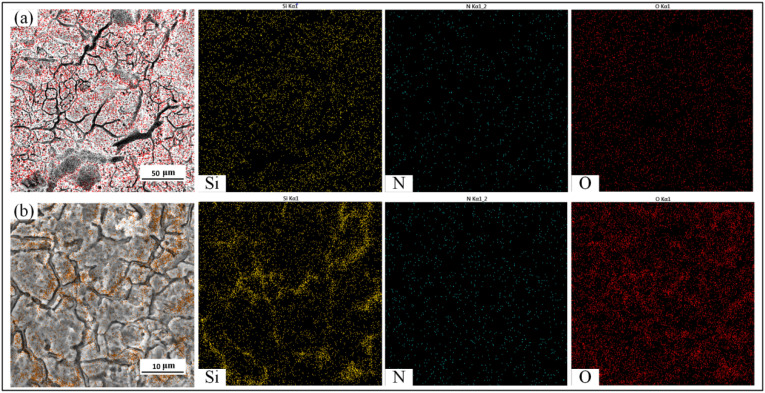
EDS results of specimens: (**a**) HEII, and (**b**) USRP+HEII.

The EDS results of the HEII specimens and USRP+HEII specimens are shown in Figure 15. Si densely and uniformly distributes in the surface of the HEII specimen, while N and O are less distributed. For the USRP+HEII specimen, Si distributes more densely in the surface. There is a more obvious aggregation phenomenon in some regions, and these regions correspond to the location of thermal cracks, which indicates that no significant loss of Si is found in the cracks. Slightly denser N is found in the surface of the USRP+HEII specimen than that of HEII specimen. It can be inferred that USRP treatment enhances the binding ability of 8407 die steel with N. Moreover, O densely distributes in the surface of the USRP+HEII specimen and is not removed via pickling during the thermal fatigue test. Thus, it can be inferred that O has a good protective effect on the specimen surface.

**Figure 16:**
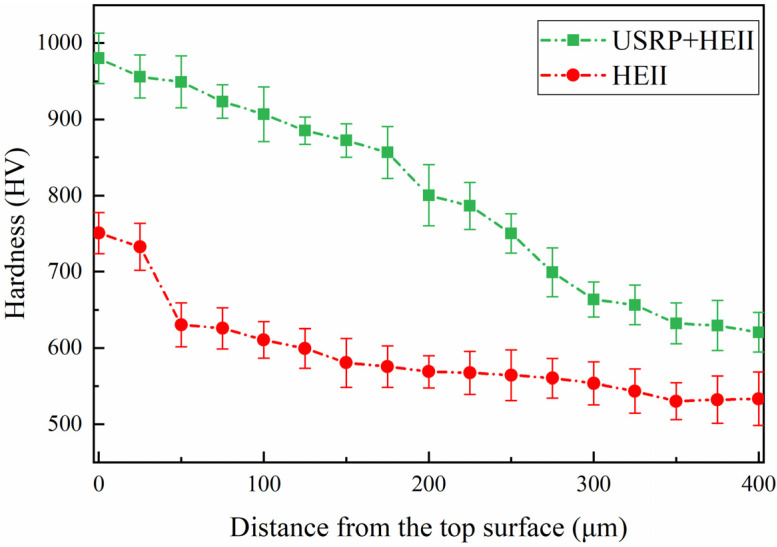
Cross-sectional hardness distribution of HEII specimens and USRP+HEII specimens after thermal fatigue.

The cross-sectional microhardness values of the HEII specimens and USRP+HEII specimens after thermal fatigue are shown in Figure 16. After thermal fatigue, the cross-sectional hardness decreases in both types of specimens. The maximum hardness of the HEII specimen is 750 HV, and decreases to a large extent in the first 50 μm from the surface. Then, the hardness shows a stable decreasing trend and maintains at 520 HV after a depth of 350 μm. The maximum hardness of the USRP+HEII specimen is 980 HV, and the overall hardness shows a stable decreasing trend. The coarse grains in the HEII specimen surface are unable to resist the heat deformation stress generated during the thermal cycle, resulting in the loss of the reinforcing phase. On the contrary, the refined grains in the surface of the USRP+HEII specimen help to resist the heat deformation stress. The elements of the strengthened phase have a stronger bonding ability with the matrix, and the strengthening effect can be maintained after several thermal cycles.

**Figure 17:**
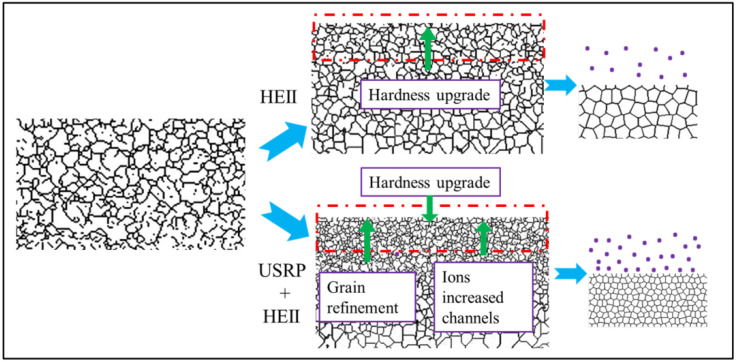
Schematic diagram of strengthening mechanism.

The schematic diagram of the strengthening mechanism for the HEII specimens and USRP+HEII specimens is shown in Figure 17. After HEII, the grains in the surface undergo a slight refinement under the high-temperature atmosphere. The ions emitted at high speed enter the surface of the material, and the alloying facilitates the enhancement of surface properties. After the USRP, the grains in the surface undergo significant refinement. The grain boundaries largely increase, providing many more channels for ion diffusion, thus further enhancing the surface properties. In addition, the high-frequency impacting on the material surface leads to an increase in non-equilibrium defects, like high-density dislocation [29], and the surface activation energy is reduced to promote the entry of activation ions into the material at these defects, providing a stable location for their presence and avoiding element loss under complex conditions. Thus, USRP pretreatment provides favorable conditions for HEII treatment with better performance against wear. In fact, the strengthening effect induced via surface plastic deformation can increase the depth of ion implantation. Thus, surface-strengthening methods based on plastic deformation can all improve the effect of ion implantation more or less. For instance, studies show that shot peening can increase the depth of ion implantation, thereby improving the fatigue properties of structural components [30].

## 4. Conclusions

The surface of 8407 die steel was first treated using ultrasonic surface rolling and then treated with high-energy ionized silicon nitride. The microstructure, chemical elements, hardness, frictional wear, and thermal fatigue properties of the strengthened surface layer were investigated and summarized as follows:

The depth of the silicon nitride-enriched layer is 37 μm and 62 μm for the HEII specimen and USRP+HEII specimen, respectively. The grain size of the USRP+HEII specimens shows a gradient increase, and the refined microstructure in the surface effectively increases the depth of high-energy ion implanting.

The surface hardness of the USRP+HEII specimen reaches 1089 HV, which is 25.6% higher than that of the HEII specimen, and the depth of the hardened layer is about 20% higher.

The USRP+HEII specimens have lower friction coefficients with oil lubrication and better wear resistance under medium- and high-frequency conditions.

The wear volumes of the USRP+HEII specimens at different test frequencies are lower than those of the HEII specimens, and the wear mechanism is mainly abrasive wear and slight adhesive wear.

The USRP+HEII specimens have shorter and shallower thermal cracks than the HEII specimens after thermal fatigue testing, and the curing effect of the implanted elements is better, which can maintain higher hardness.

The surface-strengthening technology of mold steel in this work reduces the wear and tear of the mold surface as well as surface cracks, which can effectively extend the service life of mold parts.

Considering that ultrasonic surface rolling introduces residual stresses in the mold surface, this paper is deficient in the study of the effect of residual stresses on the performance of the mold surface. Moreover, it is difficult to process the shaped surface of ultrasonic surface rolling, so there are limitations in the application scenarios.

## Figures and Tables

**Table 1:** The chemical composition of 8407 die steel (wt.%).

Element	C	Si	Mn	Cr	Mo	V	Fe
Content	0.39	1	0.4	5.2	1.4	0.9	Residual

**Table 2:** Ultrasonic surface rolling parameters.

Frequency /KHz	Amplitude /μm	Spindle Speed /r·min^−1^	Load /N	Feed Rate /mm·r^−1^
20	7.5	80	400	0.1

**Table 3:** The processing parameters of HEII.

Voltage/V	Furnace Pressure/Pa	Heat Preservation Temperature/°C	Heat Preservation Time/h
1000	20	450	80

**Table 4:** Half-height width variation of γ-Fe diffraction peak.

2θ	35.705	47.381	64.089
FWHM (HEII)	0.1929	0.0966	0.1550
FWHM (USRP+HEII)	0.3237	0.2344	0.4707

## Data Availability

Data is unavailable due to privacy.
